# The role of fluorescence angiography in colonic interposition after esophagectomy

**DOI:** 10.1093/dote/doac076

**Published:** 2022-10-29

**Authors:** J J Joosten, S S Gisbertz, D J Heineman, F Daams, W J Eshuis, M I van Berge Henegouwen

**Affiliations:** Department of Surgery, Amsterdam UMC Location University of Amsterdam, Amsterdam, the Netherlands; Department of Surgery, Amsterdam UMC location Vrije Universiteit Amsterdam, Amsterdam, the Netherlands; Cancer Center Amsterdam, Imaging and Biomarkers, Amsterdam, the Netherlands; Department of Surgery, Amsterdam UMC Location University of Amsterdam, Amsterdam, the Netherlands; Cancer Center Amsterdam, Imaging and Biomarkers, Amsterdam, the Netherlands; Department of Surgery, Amsterdam UMC location Vrije Universiteit Amsterdam, Amsterdam, the Netherlands; Cancer Center Amsterdam, Imaging and Biomarkers, Amsterdam, the Netherlands; Department of Surgery, Amsterdam UMC location Vrije Universiteit Amsterdam, Amsterdam, the Netherlands; Cancer Center Amsterdam, Imaging and Biomarkers, Amsterdam, the Netherlands; Department of Surgery, Amsterdam UMC Location University of Amsterdam, Amsterdam, the Netherlands; Cancer Center Amsterdam, Imaging and Biomarkers, Amsterdam, the Netherlands; Department of Surgery, Amsterdam UMC Location University of Amsterdam, Amsterdam, the Netherlands; Cancer Center Amsterdam, Imaging and Biomarkers, Amsterdam, the Netherlands

**Keywords:** colonic interposition, indocyanine green (ICG), esophagectomy, near infrared, fluorescence angiography

## Abstract

Colonic interposition is an alternative for gastric conduit reconstruction after esophagectomy. Anastomotic leakage (AL) occurs in 15–25% of patients and may be attributed to reduced blood supply after vascular ligation. Indocyanine green fluorescence angiography (ICG-FA) can visualize tissue perfusion. We aimed to give an overview of the first experiences of ICG-FA and AL rate in colonic interposition. This study included all consecutive patients who underwent a colonic interposition between January 2015 and December 2021 at a tertiary referral center. Surgery was performed for the following indications: inability to use the stomach because of previous surgery or extensive tumour involvement, cancer recurrence in the gastric conduit, or because of complications after initial esophagectomy. Since 2018 ICG-FA was performed before anastomotic reconstruction by administration of ICG injection (0.1 mg/kg/bolus), using the Spy-phi (Stryker, Kalamazoo, MI). Twenty-eight patients (9 female, mean age 62.8), underwent colonic interposition of whom 15 (54%) underwent ICG-FA-guided surgery. Within the ICG-FA group, three (20%) AL occurred, whereas in the non-ICG-FA group, three AL and one graft necrosis (31%) occurred (*P*=0.67). There was a change of management due to the FA assessment in three patients in the FA group (20%) which led to the choice of a different bowel segment for the anastomosis. Mean operative times in the ICG-FA and non-ICG-FA groups were 372±99 and 399±113 minutes, respectively (*P*=0.85). ICG-FA is a safe, easy and feasible technique to assess perfusion of colonic interpositions. ICG-FA is of added value leading to a change in management in a considerable percentage of patients. Its role in prevention of AL remains to be elucidated.

## INTRODUCTION

Surgical resection of the esophagus combined with a lymph node dissection is the cornerstone of esophageal cancer treatment.^1–3^ Restoration of continuity is generally achieved by the formation of a gastric conduit.^4^ The stomach is preferred because of its blood supply: due to the existence of abundant vascular anastomoses between the right and left gastric and gastroepiploic arteries, even after transection of the left gastric and left gastro-epiploic vessels, blood supply to the fundus remains substantial. Furthermore, the stomach has the ability to reach the neck to allow for a tension-free anastomosis and a gastric conduit is a less extensive operation with less anatomical changes compared with a colonic interposition.^5^

However, in certain circumstances (i.e. extensive tumour involvement of the stomach, loss, after gastrectomy), the stomach cannot be used as a conduit, in these cases a colonic interposition is the alternative of choice.^6^^,^^7^

Despite improvements in surgical technique, perioperative care and technology, the morbidity rate in colonic interpositions remains high at 60%.^8^^,^^9^ Graft necrosis, anastomotic leakage (AL) and anastomotic strictures are reported in 10–30% of patients.^9^ The high morbidity rate is attributed to the complexity of the operation, longer operating times and the additional anastomoses.

Adequate perfusion of the conduit and anastomosis is an important condition for establishing a viable anastomosis.^10^ Indocyanine green fluorescence angiography (ICG-FA) is a technique that is able to assess perfusion real-time and is proven a safe tool to show perfusion margins. Its added value in esophageal and colorectal anastomosis assessment in the elective setting is widely investigated and recognized.^11^^,^^12^

However, data regarding its feasibility and application during colonic interpositions are limited to case reports.^13–15^ In addition, to our knowledge no one has reported the utility or first experiences of ICG-FA for colonic interpositions in a case series. The aim of this study is to describe the use of ICG-FA in this patient group and postoperative anastomotic outcomes.

## METHODS

This is a cohort study of all consecutive patients who underwent colonic interposition at Amsterdam UMC from January 2015 until December 2021. Clinical data of consenting patients were prospectively collected in an electronic patient system.

Colonic interposition was performed as a primary or secondary reconstruction for a number of indications: (1) inability to use the stomach as a result of a previous gastric resection or (2) extensive tumour involvement of both stomach and esophagus, (3) oncologic recurrence in the gastric conduit requiring resection or (4) complications after initial esophagectomy with reconstruction failure and resection of the gastric conduit (i.e. ischemia/necrosis). In patients with a secondary reconstruction after several months, the colonic interposition was placed anteriorly in the mediastinum (retrosternally).

From the beginning of 2018, ICG-FA was implemented as standard procedure; patients after this time underwent ICG-FA-guided surgery.

### Surgical procedure and formation of colonic interposition

In preparation to the surgery, patients were given a mechanical bowel preparation 24–48 hours before the operation. In all patients, the right hemicolon was used, including the terminal ileum for an isoperistaltic interposition. After midline laparotomy, the omentum was dissected (if still present) and the right hemicolon was mobilized. The definitive choice of the colonic segment relied on the intraoperative evaluation of the efficiency of the right colic artery or right branch of the middle colic artery, after sequential clamping of the ileocolic artery and the arcade at dissection level of the ileum during a 30-minute clamping test with bulldog clamps. The vascular pedicles of the right colic artery and the ileocolic artery were dissected up to their origin to preserve all proximal vascular anastomoses between the main pedicles. The viability was assessed anticipating on the general aspect of the bowel and the absence of venous congestion. Other cues such as peristalsis, bleeding at the resection line and palpation of the vascular pedicle were taken into account. In the ICG-FA group, additional assessment of viability was done by using ICG-FA. When the right hemicolon was judged as viable for the use of interposition, the ileocolic artery was ligated. The colonic interposition was then pulled up to the neck through the posterior mediastinum in primary surgery or retrosternally in secondary surgery; when both these routes were not available, for example after prior sternotomy, a subcutaneous tunnel was used. To obtain sufficient reach, the colon was fully mobilized, no additional maneuvers (for instance manubrium resection) were standardly carried out. The cervical anastomosis was manually constructed between the esophagus and preferably the terminal ileum and otherwise the colon, in an end-to-end manner using a two-layered technique (in esophago-colonic anastomosis only part of the cross stapling was opened to ensure similar diameter). Further continuity was performed by a colo-jejunal anastomosis, with a Roux-Y limb, and an ileo-colonic anastomosis. Finally, the mesenteric defects were closed and a feeding jejunostomy was created. Patients were postoperatively fed by parenteral nutrition until bowel movement, then tube feeding via the jejunostomy and oral diet were gradually introduced. A nasogastric tube was placed in the colonic interposition intraoperatively to decompress the colonic interposition in the early postoperative period.

### ICG-FA and endpoints

ICG-FA was performed with Stryker (Stryker, Kalamazoo, MI, U.S.A.) imaging system: SPY Portable Handheld Imager (SPY-PHI). To create vascular contrast, a single bolus of indocyanine green (Verdye, Diagnostic Green, Aschleim) was administered intravenously. The ICG-FA video was evaluated in real time on a high-resolution monitor. Perfusion at the initially planned anastomotic site was judged adequate after ‘white light’ assessment in all patients. Decision making upon FA was based on the subjective interpretation (without the use of quantification of the fluorescent signal) of the images by the surgeon. All assessments were interpreted by the same surgeon (MvBH).

The primary endpoint was feasibility of ICG-FA in colonic interpositions. Other endpoints were the occurrence of postoperative anastomotic complications in the ICG-FA group versus the non ICG-FA group, reoperations and mortality within 90 days and change of management due to the FA. Postoperative anastomotic complications included cervical AL, graft necrosis and anastomotic stricture. Only cervical ALs are impacted by FA assessments. Cervical AL and graft necrosis were defined according to the Esophagectomy Complications Consensus Group classification and reinterventions were classified according to the Clavien–Dindo (CD) score.^16^^,^^17^ Clinically relevant benign strictures were defined as a score for dysphagia ≥2 and treatment by ≥1 dilatation. Change of management was defined as any deviation from the initial strategy, according to the result of the FA test.

### Statistics

Categorical data are presented as number of cases and percentages, whereas continuous data are shown as either mean ± standard deviation or as median and interquartile range (IQR), depending on data distribution. Length of stay in days between patients with or without ICG-FA was compared using the Mann–Whitney U test. Categorical variables were compared using a χ^2^ test. A *P*-value ≤0.05 was considered statistically significant. Data were analyzed using the Statistical Package for Social Sciences of IBM Statistics (IBM Corp., Armonk, NY, USA), version 26.0.

## RESULTS

### Patient characteristics

In total, 28 patients were included in this study with a mean age of 62.8 ± 9.9 years at time of the colonic interposition surgery. Nine patients (32%) were female. Patient characteristics are outlined in Table 1. Most patients (78%) underwent a secondary colonic interposition. The indications of the colonic interpositions are summarized in Table 2.

**Table 1:** Patient characteristics

	**ICG-FA (*n* = 15)**	**No ICG-FA (*n* = 13)**	**Total (*n* = 28)**
Sex, female	5 (33)	4 (31)	9 (32)
Age in years, mean ± SD	64.5 ± 10.8	60.9± 8.8	62.8 ± 9.9
BMI(kg/m^2^) mean ± SD	25.6 ± 3.5	23.5± 5.2	24.6 ± 4.4
ASA classification			
>2	7 (48)	2 (15)	9 (32)
Smoker			
Active	3 (20)	1 (8)	4 (14)
Stopped (<10jr)	7 (47)	8 (62)	15 (54)
No^*^	5 (33)	4 (31)	9 (32)
Pulmonary history	2 (13)	0	2 (7)
Cardiovascular history	4 (27)	2 (15)	6 (21)
Diabetes mellitus	3 (20)	0	3 (11)

Data shown in n (%) unless otherwise stated, ^*^ or stopped >10 years ago. Vascular comorbidity: brain infarction, myocardial infarction or peripheral vascular disease. Pulmonary comorbidity: asthma or COPD

ASA, American Society of Anesthesiology; BMI, body mass index; SD, standard deviation

**Table 2:** Patient characteristics of ICG FA-guided colonic interposition

**Non FA-guided primary coloplasty**						
**Patient**	**Histology**	**Tumour location**	**Reason for coloplasty**	**Neoadjuvant treatment** ^†^	**ypT**	**ypN**
1	AC	Distal esophagus	Tumour invasion into cardia	Yes, CRT	3	1
2	AC	Cardia	Tumour invasion into esophagus	Yes, CT	2	0
3	AC	Distal esophagus	previous bariatric surgery	Yes, CRT	3	0
4	AC	Cardia	Tumour invasion into esophagus	Yes, CT	3	1
**Non FA-guided secondary coloplasty**						
**Patient**	**Histology**	**Initial procedure**	**Reason for coloplasty**	**Neoadjuvant treatment** ^†^	**(y)pT**	**(y)pN**
5	AC	MI Ivor Lewis	Restoration of continuity	No	−	−
6	Carcinosarcoma	Acute THE without reconstruction due to perforation pT4aN3 tumour	Restoration of continuity	No	−	−
7	AC	Open Ivor Lewis for esophageal benign stenosis	Primary tumour in gastric conduit	Yes, CRT	2	0
8	AC	Open Ivor Lewis	Recurrence in gastric conduit	Yes, CRT	3	0
9	AC	MI McKeown	Restoration of continuity	No	−	−
10	AC	Total gastrectomy	Recurrence esophagojejunostomy	No	3	2
11	AC	Open Ivor Lewis for achalasia	Primary tumour in gastric conduit	Yes, CRT	2	0
12	SCC	MI McKeown	Restoration of continuity	No	−	−
13	AC	MI Ivor Lewis	Restoration of continuity	No	−	−
**FA-guided primary coloplasty**						
**Patient**	**Histology**	**Location**	**Reason for coloplasty**	**Neoadjuvant treatment** ^†^	**(y)pT**	**(y)pN**
14	AC	Cardia	Tumour invasion into esophagus	No	4a	3b
15	AC	Distal esophagus	Previous gastric surgery	Yes, CRT	0	0
**FA-guided secondary coloplasty**						
**Patient**	**Histology**	**Initial procedure**	**Reason for coloplasty**	**Neoadjuvant treatment** ^†^	**(y)pT**	**(y)pN**
16	AC	Open Ivor Lewis	Restoration of continuity	No	−	−
17	AC	Open Ivor Lewis because of erosion of esophagus by etchant	Primary tumour in gastric conduit	Yes, CRT	0	0
18	AC	MI Ivor Lewis	Restoration of continuity	No	−	−
19	AC	MI McKeown	Restoration of continuity	No	−	−
20	AC	MI McKeown	Restoration of continuity	No	−	−
21	AC	MI McKeown	Restoration of continuity	No	−	−
22	AC	MI McKeown	Restoration of continuity	No	−	−
23	−	Open proximal gastrectomy for incarcerated hernia diaphragmatica	Restoration of continuity	No	−	−
24	SCC	Open Ivor Lewis	Restoration of continuity	No	−	−
25	SCC	MI McKeown	Restoration of continuity	No	−	−
26	AC	Total gastrectomy	Recurrence of tumour esophagus	Yes, CT	3	0
27	AC	MI McKeown	Restoration of continuity	No		
28	Diffuse gastric AC	MI Ivor Lewis	Diffuse gastric tumour in gastric conduit	No	3	2

^†^This includes neoadjuvant treatment before the colonic interposition procedure

AC, adenocarcinoma; SCC, squamous cell carcinoma; MI, minimal invasive; GEJ, gastroesophageal junction; CT, chemotherapy; CRT, chemoradiotherapy; THE, transhiatal esophagectomy

#### Operation characteristics

More than half of the patients underwent intraoperative ICG-FA (54%). In all but one patient (96%), the ascending colon (right coloplasty) was used as interposition segment; in the remaining patient, the transverse colon with the left branch of the middle colic artery as vascular pedicle was used as colonic segment. The right colonic segment was mainly pedicled on the right branch of the middle colic artery. However, in one patient, there was not sufficient reach to obtain an anastomosis and subsequently the right branch of the middle colic artery was ligated to obtain a tension free anastomosis.

The interposition was positioned retrosternally in the anterior mediastinum in 17 patients (61%), in the posterior mediastinum in 10 patients (36%) and subcutaneously in 1 (4%) patient. Mean operative time was 385 ± 100 minutes; in the ICG-FA group and the non ICG-FA group, this was 372 ± 99 and 399 ± 113 minutes, respectively (*P* = 0.85). Median intraoperative blood loss was 400 mL (IQR 300–500) in the ICG-FA group and 435 mL (IQR 263–875) in the non ICG-FA group.

#### ICG-FA

**Fig. 1:**
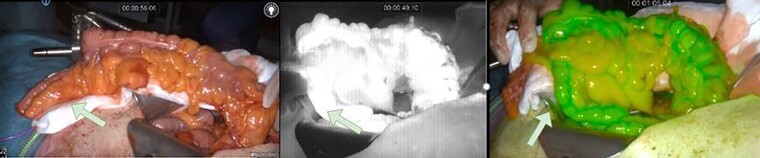
A patient with clear fluorescence enhancement on the planned anastomotic site of the colonic segment after clamping, before ligation. Right hemicolon and terminal ileum used for isoperistaltic colonic interposition. Green arrow marks the proximal anastomotic site.

**Fig. 2:**
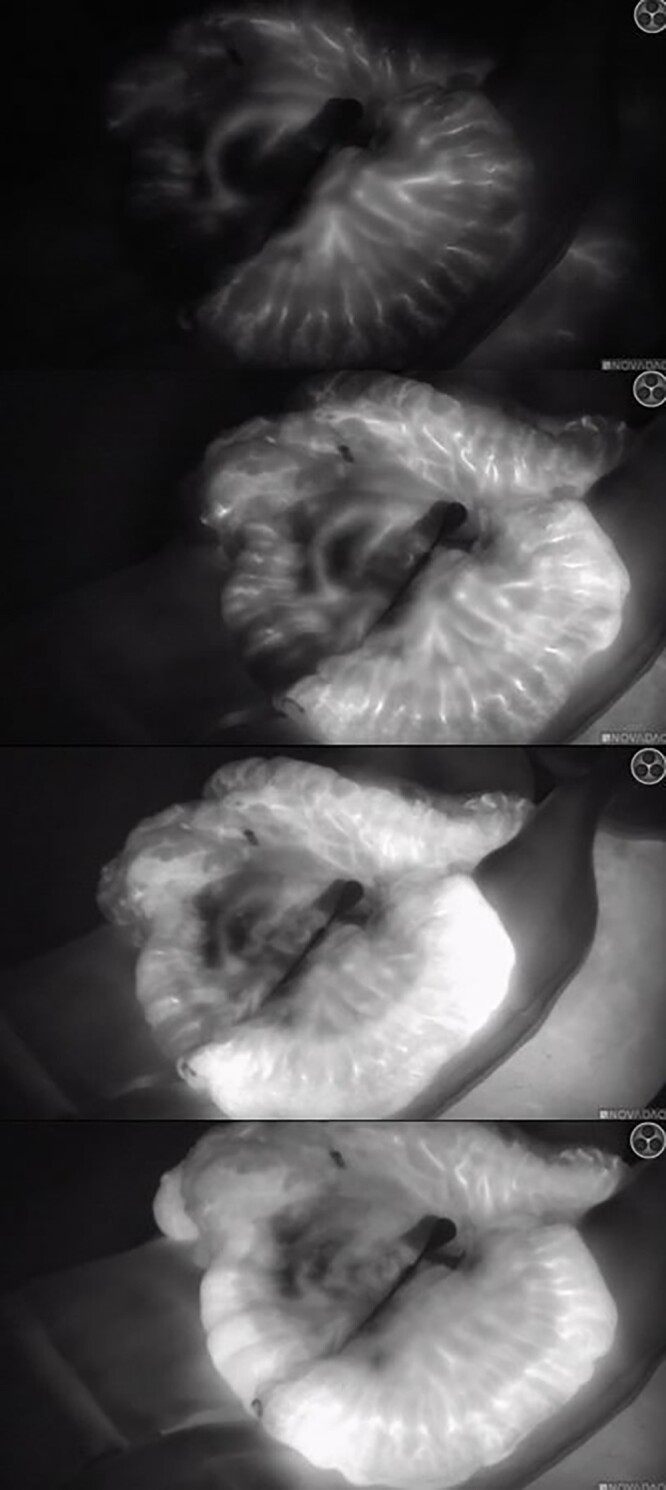
A patient with clear fluorescence enhancement of the right hemicolon (terminal ileum not visible) after clamping, before ligation. The images show fluorescent enhancement after 20, 25, 30 and 50 seconds after ICG administration.

**Fig. 3:**
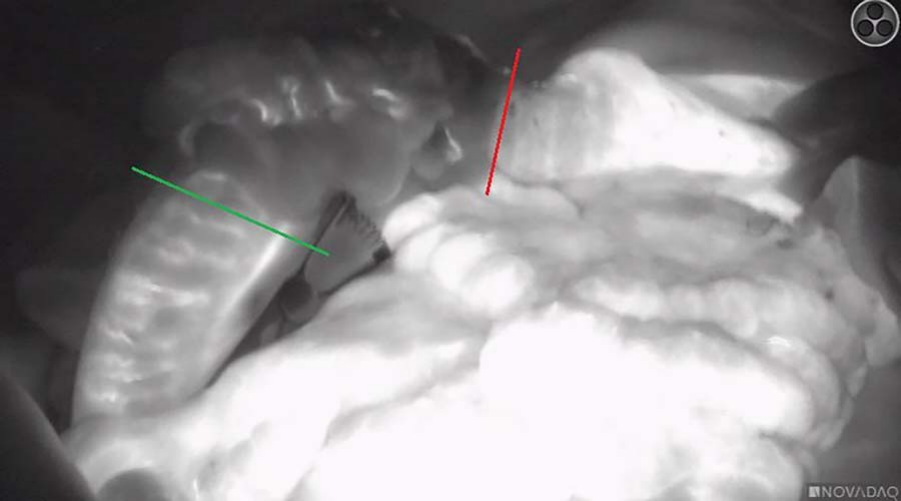
A patient with a change of management due to the ICG-FA during a right hemicolon and terminal ileum for isoperistaltic colonic interposition. The red line represents the planned proximal anastomotic site of the ileum and the green line the actual anastomotic site after the ICG-FA assessment.

ICG-FA was performed successfully in all patients with the intention on using fluorescence imaging (Figs 1 and 2), without any adverse events following ICG administration. Perfusion of the colonic interposition at the initially planned anastomotic site was judged adequate after ‘white light’ assessment in all patients. Based on the FA, a change of management was opted in three patients (20%). This change of management (Fig. 3) consisted of additionally resecting 5–10 cm length of the ileum in two patients and in one patient using the ascending colon for the anastomosis instead of the terminal ileum. One of the patients in whom 5–10 cm ileum was additionally ligated, developed an AL. During endoscopy, a small anastomotic dehiscence was observed, insufficient to place an endosponge. The cervical wound was opened, rinsed and antibiotic was intravenously administered.

#### Endpoints

Postoperative outcomes are summarized for both groups in Table 3. AL was observed in seven out of 28 patients (25%), which in one patient was secondary to graft necrosis. In the ICG-FA group, three patients (20%) had an AL, whereas in the non ICG-FA group, an AL occurred in four patients (31%) (*P* = 0.67).

**Table 3:** Postoperative outcomes of the patient groups

	**ICG-FA (*n* = 15)**	^*^CoM (n = 3)	**Non-ICG-FA (*n* = 13)**	*P*-value^**^	**Total (*n* = 28)**
AL	3 (20)	*1 (33)*	4 (31)	0.67	7 (25)
*Graft necrosis*	*0*	*0*	*1 (8)*		*1 (4)*
Anastomotic stricture	1 (7)	*0*	2 (15)	0.59	3 (11)
Management of AL					
CD ≤2	2 (13)	*1 (33)*	2 (15)		4 (14)
CD 3a	0	*0*	0		0
CD 3b	0	*0*	0		0
CD 4a	1 (7)	*2 (7)*	1 (8)		2 (7)
CD 4b	0	*0*	0	0.61	0
Other complication					
CD 2	1 (7)	*0*	2 (15)		3 (11)
CD 3a	0	*0*	0		0
CD 3b	2 (13)	*1 (33)*	0		2 (7)
CD 4a	3 (20)	*1 (33)*	4 (31)		7 (25)
CD 5	1 (7)	*0*	0	0.63	1 (4)
Total LOS, days (median (IQR))	14 (10–27)	*(18–21-25)*	17 (11–22)	0.70	16 (11–25)
LOS IC, days (median (IQR))	0 (0–3)	*(0–1-4)*	1 (1–7)	0.12	1 (0–4)
Readmission due to AL	0	*0*	2 (15)	0.55	2 (7)
90 days mortality	1 (7)	*0*	0	0.48	1 (4)

Data shown in n (%) unless otherwise stated, ^*^ CoM patients are part of the ICG-FA group^**^  *P*-value calculated between ICG-FA and non ICG- FA group.

Abbreviations: AL, anastomotic leakage; LOS, length of stay; ICU, intensive care unit; CD, Clavien–Dindo

One of the patients in the ICG-FA-group with an AL had undergone a change of management due to FA intraoperatively. The CD score was ≥4 in 7% in the ICG-FA group (*n* = 1) and 8% in the non ICG-FA group (*n* = 1).

Reoperation for AL was required in one patient in the ICG-FA group as well as one in the non-ICG-FA group. The patient in the non-ICG-FA group, a necrotic colonic interposition, was resected and subsequently an esophagostoma was created. The patient in the ICG-FA group had surgery and a new end-side cervical anastomosis was constructed.

An anastomotic stricture was observed in two patients (15%) in the non ICG-FA group and in one patient (7%) of the ICG-FA group. Mortality within 90 days was observed in one patient of the ICG-FA group. This was not related to postoperative anastomotic complications: the patient died at postoperative day 8 because of a cardiac arrest after hematemesis from the nasopharynx.

## DISCUSSION

In this study, ICG-FA-guided colonic interposition was described and the role of ICG-FA was evaluated with regard to postoperative anastomotic outcomes. ICG-FA proved to be a safe and feasible technique to assess perfusion of the colonic segment and potentially assures the surgeon in the choice of the colonic segment for the interposition. This study showed that 20% of patients had a change in management due to ICG. AL remains a frequently encountered complication, also with the use of ICG-FA. In this cohort, there was no significant difference in AL between patients operated with and without the use of ICG-FA.

Despite the extensive body of research regarding the role of ICG-FA in perfusion in colorectal and in esophageal cancer surgery investigating perfusion, there is a paucity of studies available regarding the use of ICG-FA during colonic interpositions. So far, it has only been described in case reports: Wiesel *et al.* were the first to report the technical description for assessing the perfusion of the colonic interponate with ICG-FA.^13^ Recently, a video vignette by Galema and a case study by Gupta described the feasibility of ICG-FA in colonic interposition.^14^^,^^15^ This study is the first cohort study to assess the role of ICG-FA to assess perfusion in colonic segments. The ICG-FA assessment confirmed adequate perfusion of the colonic conduit and its chosen vascular pedicle, and induced a change of management in 20% of patients, in whom a different site for the anastomosis was chosen after ICG-FA.

ICG-FA has gained widespread attention for anastomotic perfusion assessment due to its relative ease of use, low cost and good safety profile relative to other intraoperative modalities. AL secondary to perfusion restriction might be prevented by careful assessment of perfusion under guidance of ICG-FA. Recent meta-analyses in colorectal and upper gastrointestinal surgery with gastric conduit reconstruction demonstrate that standard use of ICG-FA results in a decrease in AL and graft necrosis.^18^^,^^19^ In the current study, occurrence of postoperative anastomotic complications did not differ between patients with or without ICG-FA in colonic interposition. This is certainly due to the small number of patients, but the subjective interpretation of the fluorescence angiogram may also play a role. In ICG-FA in general, there is a need for quantification methods, with quantitative values that define a threshold for adequate perfusion; this may help to standardize interpretations.

The 20% rate in change of management is higher than what has been described during gastric conduit reconstruction.^20^ This might be due to the fact that in a gastric conduit there is generally little room for additional resection of the gastric conduit, as tension on the anastomosis might increase too much to make a reliable anastomosis. During colonic interposition, there are more possibilities for additional resections without putting the colonic interponate length at risk. Of the three change of management patients, one patient developed an AL. The occurrence of AL after change of management due to ICG-FA is known and described in gastric conduit reconstruction as well. This might be caused by impaired vascularization by other factors jeopardizing the perfusion of the colonic conduit and subsequently, an AL may have been inevitable.^21^

This study has some limitations. Although it is the largest series of ICG-FA up to date, the number of included patients is still small. Collection of patient and operative data was performed retrospectively. There was also a low number of events regarding anastomotic complications. Correction for the multifactorial etiology of AL was not possible owing to a low absolute number of events. Despite these limitations, this is the first cohort study describing a consecutive series of patients, with the best available evidence up till now regarding the safety and feasibility and treatment consequences of ICG-FA, and the AL rate in ICG-FA-guided colonic interposition. The ICG-FA interpretation in this study was based on the surgeon’s own judgement, and is therefore subjective. Recently it is has been demonstrated that there is a considerable inter-user variation in interpretation of FA during esophagostomy with gastric conduit reconstruction.^22^ However, this has not been assessed during reconstructions with the colonic conduit. In this study, FA was assessed by the same surgeon. However, this might have been different by someone else. There are no thresholds to guide whether the perfusion as assessed by FA is sufficient or not. Further work on ICG-FA in colonic interpositions should focus on gaining more experience with the technique and its applications, and quantification of the perfusion, thereby establishing a threshold for adequate perfusion of the colonic conduit.

In conclusion, ICG-FA is a safe, easy and feasible technique to assess perfusion of colonic interpositions. ICG-FA is of added value, leading to a change in management in a considerable percentage of patients. Its role in prevention of AL remains to be elucidated.
